# Necroptosis regulates tumor repopulation after radiotherapy via RIP1/RIP3/MLKL/JNK/IL8 pathway

**DOI:** 10.1186/s13046-019-1423-5

**Published:** 2019-11-09

**Authors:** Yiwei Wang, Minghui Zhao, Sijia He, Yuntao Luo, Yucui Zhao, Jin Cheng, Yanping Gong, Jianzhu Xie, Yulan Wang, Binjie Hu, Ling Tian, Xinjian Liu, Chuanyuan Li, Qian Huang

**Affiliations:** 1 Cancer Center, Shanghai General Hospital, Shanghai Jiao Tong University School of Medicine, 650 Xinsongjiang Road, Songjiang District, Shanghai, 201620 China; 2 Institute of Translational Medicine, Shanghai General Hospital, Shanghai Jiao Tong University School of Medicine, Shanghai, China; 3 Department of Dermatology, Duke University Medical Center, Box 3135, Durham, North Carolina 27710 USA

**Keywords:** Radiotherapy, Necroptosis, Tumor repopulation, RIP1/RIP3/MLKL/JNK/IL-8 pathway

## Abstract

**Background:**

Tumor cell repopulation after radiotherapy is a major cause for the tumor radioresistance and recurrence. This study aims to investigate the underlying mechanism of tumor repopulation after radiotherapy, with focus on whether and how necroptosis takes part in this process.

**Methods:**

Necroptosis after irradiation were examined in vitro and in vivo. And the growth-promoting effect of necroptotic cells was investigated by chemical inhibitors or shRNA against necroptosis associated proteins and genes in in vitro and in vivo tumor repopulation models. Downstream relevance factors of necroptosis were identified by western blot and chemiluminescent immunoassays. Finally, the immunohistochemistry staining of identified necroptosis association growth stimulation factor was conducted in human colorectal tumor specimens to verify the relationship with clinical outcome.

**Results:**

Radiation-induced necroptosis depended on activation of RIP1/RIP3/MLKL pathway, and the evidence in vitro and in vivo demonstrated that the inhibition of necroptosis attenuated growth-stimulating effects of irradiated tumor cells on living tumor reporter cells. The JNK/IL-8 were identified as downstream molecules of pMLKL during necroptosis, and inhibition of JNK, IL-8 or IL-8 receptor significantly reduced tumor repopulation after radiotherapy. Moreover, the high expression of IL-8 was associated with poor clinical prognosis in colorectal cancer patients.

**Conclusions:**

Necroptosis associated tumor repopulation after radiotherapy depended on activation of RIP1/RIP3/MLKL/JNK/IL-8 pathway. This novel pathway provided new insight into understanding the mechanism of tumor radioresistance and repopulation, and MLKL/JNK/IL-8 could be developed as promising targets for blocking tumor repopulation to enhance the efficacy of colorectal cancer radiotherapy.

## Background

Radiotherapy (RT) is one of the major therapeutic modalities for cancer. It is noteworthy that more than 50% of cancer patients received RT in the course of their disease treatment, and in which 40% can be cured by RT [1–3]. However, tumor repopulation remains one of the critical factors of therapeutic failure [4]. Tumor repopulation is a process that a few surviving tumor cells proliferate during or after radiotherapy even in accelerated paces. Numerous studies are dedicated to explore the molecular mechanisms of this process, and a few growth-related signal pathways, cancer stem cells and tumor microenvironment such as hypoxia, tumor educated macrophages or fibroblasts are reported to be implicated [5–7].

Apoptosis and necrosis have been recognized as positive processes for cancer treatment. However, our previous studies demonstrated that apoptosis and necrosis might facilitate tumor growth by activating proliferation signal pathway and producing growth factors during cell dying. For example, activation of apoptosis associated Caspase-3 after irradiation resulted in calcium-independent phospholipase A2 (iPLA2) cleavage and activation, which increased arachidonic acid (AA) synthesis and subsequent prostaglandin E2 (PGE2) production and release. The PGE2 released by apoptotic tumor cells then stimulated proliferation of survived tumor cells as well as angiogenesis. We named this novel tumor repopulation mechanism as “Phoenix Rising” [8]. We also found that high mobility group box 1 (HMGB1), released by irradiated necrotic tumor cells, participated in tumor repopulation [9].

Besides apoptosis, a novel programmable form of necrosis called necroptosis has recently gained attention. The activation of receptor interacting protein 3 (RIP3) and mixed lineage domain-like protein (MLKL) are both considered as the biomarkers for necroptosis [10–12]. TNF/TNF receptor 1 mediated signaling pathway is one of the most extensively studied models of necroptosis and it exists widely in different types of tumors and other pathophysiologic conditions. During TNF/TNFR1-induced necrosis, receptor interacting protein 1 (RIP1) is activated first by phosphorylation, which in turn activates RIP3 through its kinase activity and together they form the RIP1/RIP3 complex [13–16], which result in phosphorylation of MLKL. The phosphorylated MLKL, which is recognized as a biomarker for TNF-driven necroptosis, is then transported into the nucleus and to the cell membrane, eventually triggering cellular membrane rupture and cell death [17]. Accordingly, chemical inhibitors of RIP1, for example, Necrostatin-1 (Nec-1), can specially inhibit TNF-driven necroptosis [18]. It has been reported that necroptosis exists in radiation-induced cell death in endocrine cancer [19, 20]. However, the role of the necroptosis in radiation-related cancer therapy is not clearly understood. In particular, whether and how this novel form of cell death participates in radiation-related tumor repopulation is unclear.

In the present study, we hypothesized that radiation-induced necroptosis might also play an important role in tumor repopulation during radiotherapy. We provided evidence that radiation-induced necroptosis is dependent on sequential activation of RIP1/RIP3/MLKL, and necroptosis contributes to tumor repopulation through the MLKL/JNK/IL-8 axis. Furthermore, the elevated expression of IL-8 in tumor tissue is associated with a worse prognosis in colorectal cancer patients. Our data therefore suggest that the blockage MLKL/JNK/IL-8 during conventional radiotherapy might enhance the efficacy of radiotherapy in colorectal cancer.

## Materials and methods

### Cell culture and irradiation treatment

Human colorectal cancer cell line HT29, SW480 and HCT116 were obtained from Chinese Academy of Sciences Cell Bank (Shanghai, China). HT29 and HCT116 were cultured in high-glucose Dulbecco’s Modified Eagle’s Medium (DMEM) (Life Technologies) supplemented with 10% FBS and 1% penicillin/streptomycin (Life Technologies). SW480 were cultured in RPMI-1640 (Life Technologies) containing 10% FBS and 1% penicillin/streptomycin (Life Technologies). The cultured cells were kept at 37 °C in a humidified 5% CO_2_ atmosphere. The cells or mice carrying xenograpt tumor were irradiated using an Oncor linear accelerator (Siemens, Amberg, Germany) and the dose rate was 3.6 Gy/min.

### Gene transduction

The pLEX lentiviral vector system (Open Biosystem, Huntsville, AL, USA) was used to transduce exogenous genes into target cells as previous described. The firefly luciferase (Fluc) and green fluorescent protein (GFP) fusion gene was used to genetically label reporter tumor cells. The shRNA targeting RIP1, RIP3 (pGIPZ shRNA vector) or MLKL (PLKO.1 shRNA vector) were commercially available. HT29 Fluc, SW480 Fluc, HCT116 Fluc, HT29 sh-RIP1, HT29 sh-RIP3, HT29 sh-MLKL and HCT116 sh-RIP1 cells were constructed through lentivirus infection and subsequent puromycin selection at 2–3 μg/ml.

### Clonogenic formation assay

HT29, SW480 and HCT116 cells were pre-treated with or without Nec-1 (HT29 and HCT116 cells at 50 μM and SW480 cells at 80 μM) for 24 h and seeded in 6-cm dishes in triplicate in different numbers according to irradiation dose they had accepted. About 24 h later, the plates were exposed to various doses of radiation (0 Gy, 2 Gy, 4 Gy, 6 Gy, 8 Gy and 10 Gy with 100, 200, 1000, 2500, 10,000 and 50,000 cells per plate respectively). After 48 h, fresh medium was added. The plates were fixed and stained with crystal violet 10–14 days later. And the colonies with more than 50 cells were counted for survival assay.

### Flow cytometric analysis of apoptosis, necroptosis, and ferroptosis

Cell death was assessed by an Accuri C6 Flow cytometer (BD Biosciences, CA, USA). Cells pre-treated with or without Nec-1 (HT29 and HCT116 cells 50 μM, SW480 cells 80 μM), Z-vad-fmk (HT29 and SW480 cells 10 μM) and Liprosxtatin-1 (HT29 and SW480 cells 1 μM) were seeded in 6-well plates and then irradiated with 10 Gy radiation. After 3 days, the cells were washed, trypsinized, resuspended in binding buffer, and then stained for 15 min by Annexin V-FITC and propidium iodide (PI) (BD Pharmingen, CA, USA). The experiments were performed in triplicate. The criteria for distinguishing various forms of cell death: Necroptosis, the inhibition of PI positive cells by Nec-1; Apoptosis, the inhibition of Annexin V positive cells by Z-vad-fmk; Ferroptosis, the inhibition of Annexin V negative/PI negative cells by Liproxstatin-1.

### Cell death assay

Cell death was evaluated by quantifying the concentrations of lactate dehydrogenase (LDH) in the culture medium. The cells were plated in 96-well plates at a density of 5000 cells per well in triplicate and then irradiated with 10 Gy X-ray. After 3 days, the supernatants were collected and LDH release was quantified using the LDH cytotoxicity assay kit (Beyotime, China) according to the manufacturer’s recommendations.

### Cell viability assay

The cells were seeded in 96-well plates with no irradiation and 10 Gy radiation. The cell viability in triplicate was measured daily for continuous 6 days according to absorbance of the sample at 450 nm by using Cell Counting Kit (CCK-8) (Yeasen, China) on a microplate reader (Thermo Scientific, USA).

### Western blot analysis

Tumor cells were lysed on ice with ice-cold radioimmunoprecipitation (RIPA) buffer containing a protease inhibitor cocktail (Sigma-Aldrich) for 30 min. The tumor tissues in RIPA buffer were treated by ultrasound. Then, soluble protein concentrations in lysate were determined by using a BCA protein assay kit (Thermo Scientific, Fremont, CA, USA). Western blot analysis was performed mainly as previously described [21]. The following antibodies were used: caspase-3, cleaved caspase-3, caspase-8, cleaved caspase-8 (9662S, 9661S, 4790S, 9496S respectively, Cell Signaling Technology, Danvers, MA, USA), RIP1 (610,458, BD Biosciences), pRIP1 (65746S, Cell Signaling Technology, Danvers, MA, USA), RIP3 (13,526, Cell Signaling Technology, Danvers, MA, USA), pRIP3 (ab209384, Abcam, Cambridge, UK), MLKL (ab184718, Abcam, Cambridge, UK), pMLKL (ab187091, Abcam, Cambridge, UK), JNK (9252, Cell Signaling Technology, Danvers, MA, USA), pJNK (4668, Cell Signaling Technology, Danvers, MA, USA), GAPDH (5174S, Cell Signaling Technology, Danvers, MA, USA) and corresponding secondary antibodies (Jackson ImmunoResearch, PA, USA).

### Immunofluorescence analysis

Cells were seeded on dishes with glass for confocal image. After incubation at 37 °C for 24 h, the cells were exposed to 10 Gy X-ray, cultured for 48 h and then were fixed with 4% PFA paraformaldehyde for immunofluorescence staining as previously described [8]. The primary antibody was pMLKL (Abcam), and cell nuclei were counterstained with DAPI (Vector Laboratories, Burlingame, CA). Images were acquired with a confocal microscope (Leica, Germany).

### Co-immunoprecipitation

HT29 cells pre-treated with or without Nec-1 (50 μM) for 12 h were exposed to 0 Gy or 10 Gy X-ray. Equal amounts of protein were incubated with protein A/G agarose beads (Santa Cruz Biotechnology, Inc.) and anti-RIP1 or anti-RIP3 antibodies overnight at 4 °C. The beads were washed at least five times with lysis buffer, and then boiled in SDS sample buffer for further western blotting analysis.

### Tumor repopulation models and bioluminescence imaging

In vitro tumor repopulation model was simulated as followed. A small number (100–500) of firefly luciferase (Fluc)-labeled living colon cancer cells (for example, HT29-Fluc, described as reporter cells) were seeded onto a larger number (1 × 10^5^) of unlabeled lethally irradiated (10 Gy) colon cancer cells (for example, HT29 cells, described as feeder cells). The same number of reporter cells (100–500) alone or with (1 × 10^5^) of unlabeled non-irradiated tumor cells were used as control. The culture medium was 2% FBS DMEM and replaced every 2 days. After a co-culture period of 7 to 10 days, the number of labeled living cells (reporter cells) was measured via bioluminescence imaging by adding D-luciferin (bc219; Synchem UG & Co. KG, Felsberg/Altenburg, Germany) in PBS at a concentration of 0.15 mg/ml and incubation for 5 min.

In vivo tumor repopulation model was simulated by subcutaneous co-injection of a small number (5 × 10^5^) of Fluc-labeled tumor cells alone or together with a large number (5 × 10^6^) of unlabeled lethally irradiated (10 Gy) tumor cells in the hind legs of the nude mice. The growth of the Fluc-labeled cells in vivo was monitored through bioluminescence imaging two-three times a week after mice received 150 mg/kg D-luciferin in PBS (30 mg/ml) by intraperitoneally injection and 10 min reaction time.

Bioluminescence imaging machines used in this study were IVIS Lumina Series III (PerkinElmer, USA). After images were taken, the manufacturer-supplied software was used to process the images for quantitative data.

### In vivo tumorigenicity

For the tumorigenicity assay, we utilized 5-week old nude mice. One hundred microliter PBS containing a total of 5 × 10^6^ vector-transduced or MLKL shRNA-transduced HT29 cells was injected subcutaneously into either hind leg, respectively. Tumor sizes were measured 2–3 times a week using calipers, and tumor volume (V) was calculated using the formula: V = 0.5 × length×width^2^.

To evaluate the levels of pMLKL and IL-8 in tumor after radiotherapy, we injected 5 × 10^6^ HT29 cells subcutaneously into nude mice. When tumor volume reached 800mm^3^, they were exposed to 10 Gy X-ray. After 48 h, the mice were sacrificed by cervical dislocation. Tumors were dissected and stored in liquid nitrogen or fixed in 10% formalin for further analysis.

### Immunohistochemistry analysis

Immunohistochemistry analysis was conducted as previously described [22]. An EnVision™ III Detection System (GK500705; Gene Tech, Shanghai, China) was used during the process. The primary antibody included anti-IL-8 (Abcam). The images were captured using a Leica microscope.

We conducted the immunohistochemical (IHC) staining with the IL-8 antibody in a tumor microarray (TMA), which included 71 human colorectal cancer tissues. Additional file 1: Table S1 shows the patient characteristics. Two blinded independent pathologists, who had no knowledge of patient data and tumor characteristic, evaluated the IHC staining. The IL-8 staining was scored by two variables, one was the percentage of cells with stained cells (none = 0, 1–40% = 1, 41–75% = 2, and > 75% = 3), and the other was the intensity of the staining (none = 0, low = 1, moderate = 2, and strong = 3). The product of the two variables was considered as the immunoreactivity score (IS). Finally, the patients were divided into IL-8-low (IS< 2) and IL-8-high (IS ≥2) groups by IS values.

### Automated chemiluminescent immunoassays

The six cytokines in cell culture supernatants were measured according to manufacturer’s instruction using IMMULITE 1000 (SIEMENS, Germany). All samples were a pool of three samples and measurements were repeated three times.

### Other drugs

Nec-1, GSK’872, NSA, SP600125, reparixin, Z-vad-fmk and Liprosxtatin-1 were bought from Selleck Chemicals (Houston, TX, USA). Anti-IL-8 antibody and recombinant human IL-8 Protein were bought from R&D Systems.

### Statistical analysis

Statistical analysis was conducted using GraphPad Prism 6 (GraphPad Software, USA). All data were presented as the mean ± SEM (standard error of the mean). Two-tailed Student’s t-test was used for the two-group test, and one-way analysis of variance (one-way ANOVA) was used for the multi-group test. Survival analysis was conducted using the Kaplan–Meier method. A value of *p* < 0.05 was considered indicative of statistical significance.

## Results

### Necroptosis and apoptosis are independent cell death pathway

**Fig. 1:**
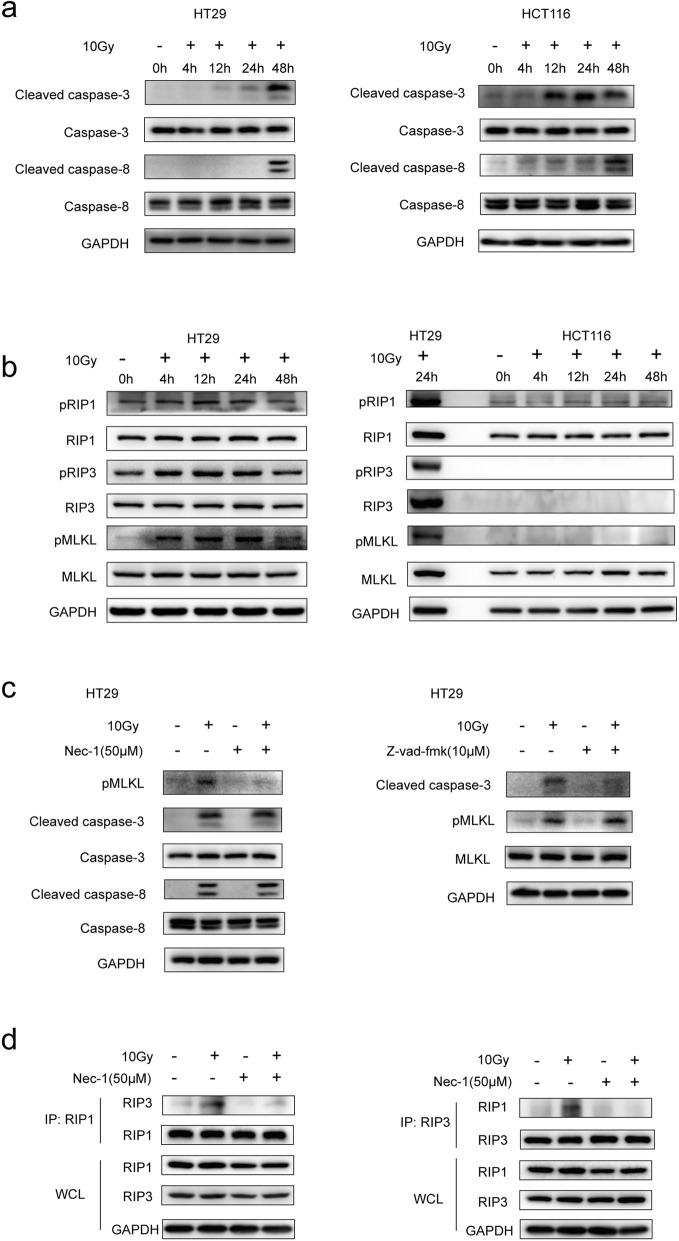
Western blot analysis of apoptosis and necroptosis associated proteins in colorectal cancer cell lines. **a** The full length and cleaved caspase-3 and 8 level at different time points post 10Gy irradiation in HT29 and HCT116 cells were evaluated by western blot. **b** The RIP1/pRIP1 (S166), RIP3/pRIP3(S227), MLKL/pMLKL(T357/S358) level at different time points post 10Gy irradiation in HT29 and HCT116 cells were evaluated by western blot. **c** Left panel, pMLKL, full length and cleaved caspase-3 and 8 level in HT29 cells at 48 h post 10Gy irradiation and pre-treatment with or without 50 μm Nec-1. Right panel, the cleaved caspase-3 and MLKL/pMLKL in HT29 cells at 24 h post 10Gy irradiation and pre-treatment with or without 10 μm Z-vad-fmk. **d** Co- immunoprecipitation of RIP1/RIP3 showed the formation of RIP1/RIP3 complex in irradiated HT29 cells and pre-treatment with or without 50 μm Nec-1. The endogenous RIP1 and RIP3 expression were determined using whole-cell lysates (WCL)

Consistent with our previous studies, we conducted western blot analysis to determine the extent of caspase-3 and caspase-8 activation after radiation. We could see that caspase-3 and caspase-8 were cleaved in HT29 and HCT116 cells after radiotherapy, indicating that radiation could induce apoptosis in these cell lines (Fig. 1a). It is interesting to note that cleaved caspsae-8 in HT29 cells begins to appear at 48 h after radiation, whereas in HCT116 cells, cleaved caspase-8 appeared as early as 4 h after radiation. To examine whether necroptosis involved in the process, we first evaluated the levels of RIP1, RIP3 and MLKL endogenous expression in HT29 and HCT116 cells by use of western blot analysis. Results showed that all three proteins were detected in HT29 cells (Additional file 1: Figure S1a). And western blot analysis revealed that pRIP1(Ser166), pRIP3(S227) and pMLKL (T357/S358) began to increase at 4 h after radiation in HT29 cells, peak at 24 h and decline at 48 h while there was no RIP3 and pRIP3 detected in HCT116 cells (Fig. 1b). Interestedly, no pMLKL had been detected in irradiated HCT116 cells although MLKL expression was seen in HCT116 cells (Fig. 1b).

To examine the relationship between radiation induced apoptosis and necroptosis, we performed western blot (Fig. 1c) to detect pMLKL in 10Gy-irratiated and pretreated with z-vad-fmk HT29 cells (pan-caspase inhibitor, 10 μM), and the cleaved caspase-3 and cleaved caspase-8 in 10Gy-irratiated and pretreated with Nec-1 HT29 cells (necroptosis inhibitor, 50 μM). The results revealed that z-vad-fmk had no influence on the level of pMLKL in irradiated cells, nevertheless Nec-1 also did not show influence on the level of cleaved caspase-3 and cleaved caspase-8 in irradiated cells.

Additionally, we performed co-immunoprecipitation experiments to elucidate the role of RIP1 and RIP3 in radiation-induced necroptosis. Figure 1d revealed that RIP1 could interact with RIP3 in irradiated HT29 cells, but which could be abrogated by Nec-1. Furthermore, Nec-1 pretreatment could reverse the radiation-induced increase of pMLKL (Additional file 1: Figure S1b). These results suggested that phosphorylation of RIP1/RIP3/MLKL and cleavage of caspase-3 and 8 occurred in HT29 and SW480 cells after irradiation (Fig. 1a-c and Additional file 1: Figure S1c), in which phosphorylation of RIP1/RIP3/MLKL occurred earlier than cleavage of caspase-8. HCT116 cells showed cleavage of caspase-3 and 8 but no phosphorylation of RIP3/MLKL because lack of RIP3 expression. Besides, the formation of RIP1 and RIP3 complex and phosphorylation of downstream factor MLKL are required for necroptosis. Finally, necroptosis and apoptosis were independent cell death pathway occurred in irradiated cells.

### Nec-1 inhibits radiation-induced necroptosis rather than apoptosis in vitro

**Fig. 2:**
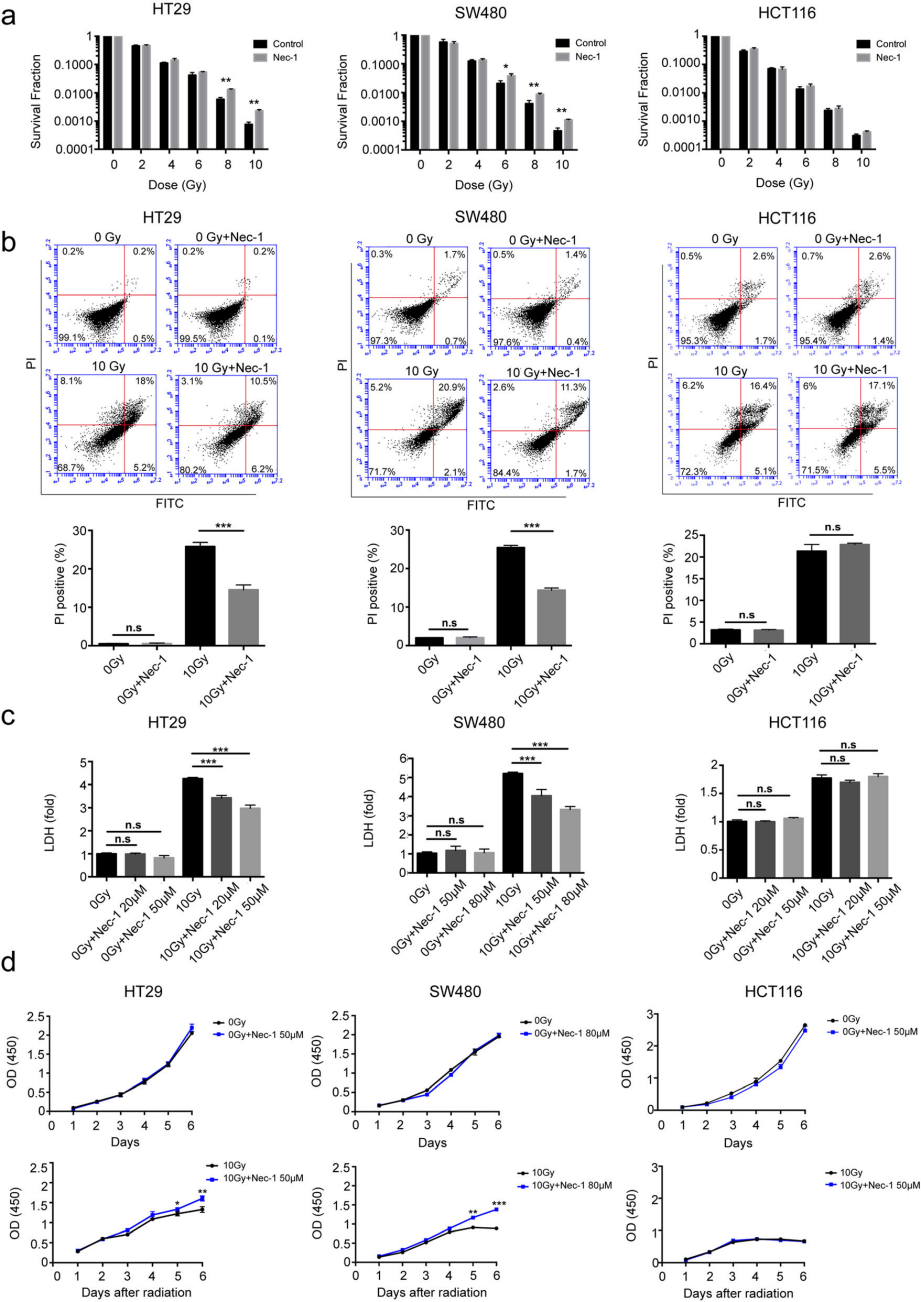
Nec-1 inhibits radiation-induced necroptosis rather than apoptosis in vitro. (a-d) The colorectal cancer cell lines (HT29, SW480 and HCT116) were pre-treated with or without Nec-1 for 24 h prior to radiation. **a** Clonogenic survival assays of irradiated HT29, SW480 and HCT116 cells were performed as indicated, student’s t-test, *n* = 3. **b** Representative graphs and statistical analysis of flow cytometry using Annexin V/PI double staining showed the cell death manner of HT29, SW480 and HCT116 after 0 Gy or 10 Gy irradiation. Percentage decreased PI positive cells by Nec-1counted as necroptosis, one-way ANOVA, *n* = 3. **c** LDH was quantified in cell culture supernatants of HT29, SW480 and HCT116 with or without 10 Gy radiation, one-way ANOVA, *n* = 3. **d** Cell viability of HT29, SW480 and HCT116 cells were assessed using a CCK-8 assay with no irradiation (upper panel) or after 10 Gy radiation (lower panel), student’s t-test, *n* = 3. n.s = not significant, * < *p* 0.05, ** *p* < 0.005, *** *p* < 0.001

Three human colorectal cancer cell lines (HT29, SW480 and HC116) were used to investigate the cellular survival fraction after radiation (0–10 Gy) with or without pretreatment of Nec-1 (the inhibitor of necroptosis confirmed above) by performing clonogenic formation assays. The results showed that radiation significantly eradicated colony forming abilities of the cells in a dose-dependent manner, which could be partly rescued by Nec-1 in HT29 and SW480, but not HCT116 cells (Fig. 2a and Additional file 1: Figure S2). Next, as indicated in Fig. 2b, flow cytometry analysis showed that the number of necrotic cells (PI-positive cells) induced by 10 Gy radiation could be reduced from 26.1 to 13.6% and from 26.1 to 13.9% after Nec-1 pretreatment in HT29 and SW480, respectively, while no obvious effect was observed in HCT116. Furthermore, we found that 10Gy-radiation resulted in killing of HT29 and SW480 via preferential stimulation of necroptosis (Additional file 1: Figure S3). In support of this conclusion, lactate dehydrogenase (LDH), a marker of cell death, was profoundly inhibited by the pretreatment of Nec-1 in 10 Gy X-ray irradiated HT29 and SW480 cells but not in HCT116 cells (Fig. 2c). As shown in Fig. 2d, CCK8 assays demonstrated that the cell viability was significantly decreased in HT29 and SW480 after 10 Gy X-ray, which was rescued by pretreatment of Nec-1, while no obvious effect was observed in HCT116. Collectively, these results demonstrated that radiation can induce necroptosis in HT29 and SW480 cell lines, which can be reversed by Nec-1, while necroptosis was not a significant factor in radiation induced killing of HCT116 cells.

### Radiation-induced necroptosis depended on the RIP1/RIP3/MLKL signaling pathway

**Fig. 3:**
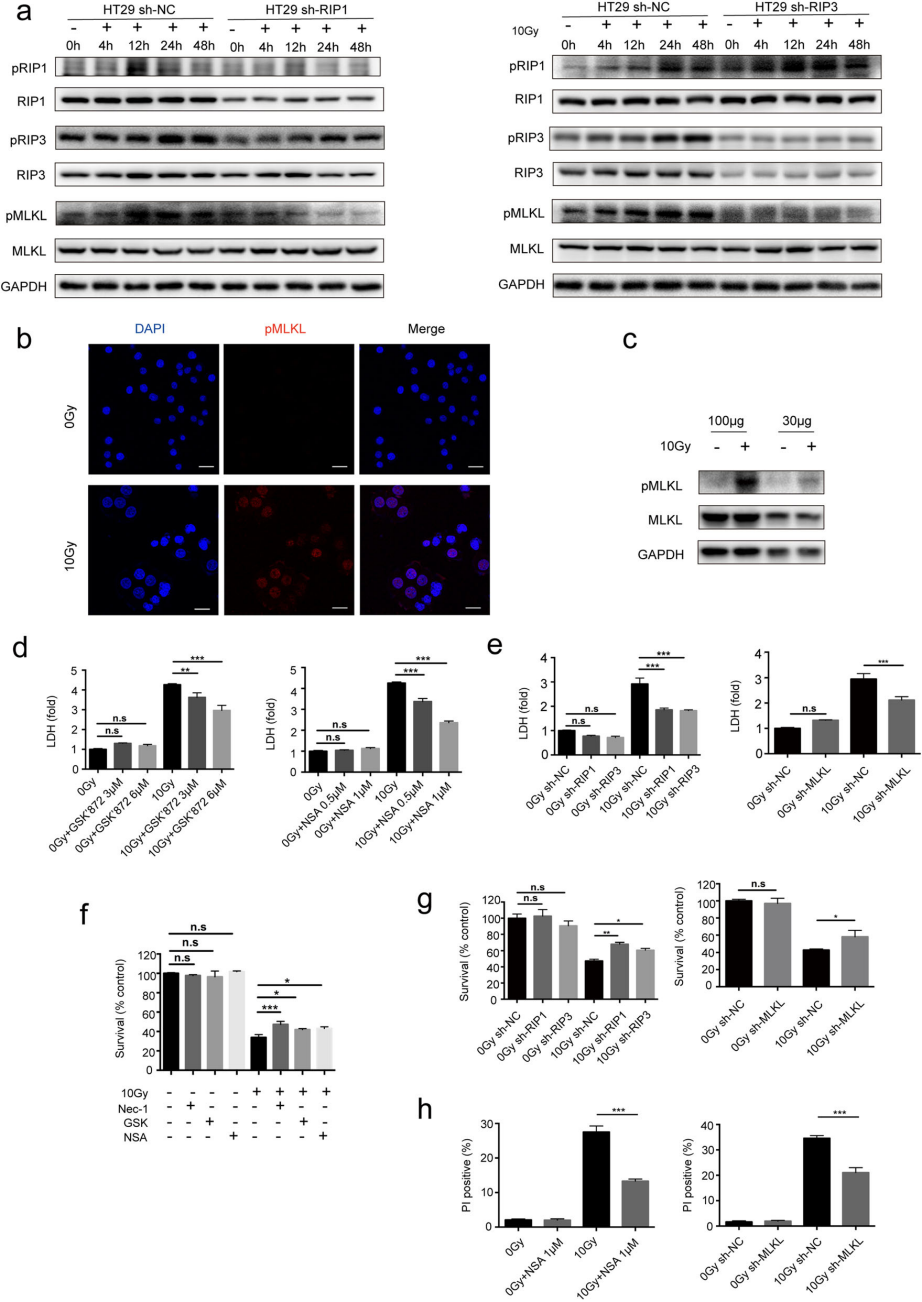
Radiation-induced necroptosis depended on the RIP1/RIP3/MLKL signaling pathway. **a** Left panel, the level of pRIP1(S166), pRIP3(S227), pMLKL (T357/S358) and corresponding endogenous protein expression in RIP1 knockdown and vector-transfected HT29 cells at different time point post10 Gy-irradiation were evaluated by western blot. Right panel, the pRIP1/pRIP3/pMLKL and corresponding endogenous protein expression in RIP3 knockdown and vector-transfected HT29 cells treated like left panel. **b** Confocal immunofluorescence analysis showed increased pMLKL expression in 10 Gy irradiated HT29 cells than non-irradiated cells. Scale bar: 25 μm. **c** The pMLKL level in the HT29 xenograft tumor samples 48 h post 10Gy-irradiation or non-irradiation. Please note 30 μg and 100 μg were loaded on same blot. **d** LDH release were quantified in cell culture supernatants from HT29 cells pretreated with the inhibitor of RIP3 (left panel, GSK’ 872) or MLKL (right panel, NSA) for 24 h with 10 Gy irradiation or non-irradiation, one-way ANOVA, *n* = 3. **e** LDH release were quantified in cell culture supernatants from HT29 sh-NC cells, HT29 sh-RIP1 cells, HT29 sh-RIP3 cells and HT29 sh-MLKL cells treated with or without 10 Gy radiation, one-way ANOVA, *n* = 3. **f** Cell viability was performed through CCK-8 assay in HT29 cells pretreated with the inhibitor of RIP1 (Nec-1), RIP3 (GSK’ 872) or MLKL (NSA) and 6 days later after 10Gy irradiation or non-irradiation, one-way ANOVA, *n* = 3. **g** Cell viability was performed through CCK-8 assay 6 days later in HT29 sh-NC cells, HT29 sh-RIP1 cells, HT29 sh-RIP3 cells and HT29 sh-MLKL cells with or without 10 Gy irradiation, one-way ANOVA, *n* = 3. **h** The statistical analysis of Annexin V/PI stain cells by flow cytometry. Left panel, the percentage of PI positive in 10Gy-irradiated or non-irradiated HT29 cells which were pretreated with or without MLKL inhibitor (NSA). Right panel, the percentage of PI positive in HT29 sh-NC or sh-MLKL cells with non-irradiation or 10 Gy irradiation. Necroptotic cells were those decreased PI positive cells by Nec-1, one-way ANOVA, *n* = 3. n.s = not significant, * < *p* 0.05, ** *p* < 0.005, *** *p* < 0.001

In order to elucidate the role of RIP1/RIP3/MLKL during radiation induced necroptosis, we manipulated the expression or function of RIP1/RIP3/MLKL by using genetic knock-down technology or small molecules. As shown in Fig. 3a, the increase in pMLKL was markedly inhibited in 10 Gy irradiated HT29 sh-RIP1 cells and HT29 sh-RIP3 cells, compared with irradiated parental HT29 cells (Additional file 1: Figure S4a). These findings demonstrate that MLKL is a downstream regulator of the RIP1/RIP3 complex after radiation. Moreover, Fig. 3b showed that more pMLKL could be detected in HT29 cells after 10 Gy irradiation by immunofluorescence microscopy. To further evaluate whether 10 Gy irradiation can induce the activation of MLKL in vivo, we examined the level of pMLKL using a mouse xenograft tumor derived from HT29 cells. Consistent with the in vitro results, western blot assay of tumor tissue showed that radiation could significantly upregulate the levels of pMLKL in tumor in vivo (Fig. 3c)*.*

Next, we investigated whether the RIP1/RIP3/MLKL pathway plays a pivotal role in necroptosis in 10 Gy-irradiated HT29 cells. As shown in Fig. 3d, pretreatment with GSK’872 (a RIP3 inhibitor) and Necrosulfonamide (an MLKL inhibitor) significantly abolished HT29 cell death caused by radiation. Similar results were also observed in RIP1, RIP3 and MLKL knockdown cells respectively (Fig. 3e). Furthermore, inhibitors of RIP1, RIP3 and MLKL as well as the genetic suppression of RIP1, RIP3 and MLKL could also partially reverse the inhibition of cell viability caused by radiation in HT29 cells (Fig. 3f and g). In contrast, there was no effect on cell death in HCT116 sh-RIP1 cells, compared with parental HCT116 cells (Additional file 1: Figure S4b). Additionally, NSA pretreatment or knock-down of MLKL inhibited the number of necrotic cells (PI-positive cells) in HT29 cells after 10 Gy radiation analyzed by flow cytometry (Fig. 3h and Additional file 1: Figure S4c). Collectively, these results imply that radiation induces necroptosis through the activation of the RIP1/RIP3/MLKL signaling pathway.

### The blockade of the RIP1/RIP3/MLKL signaling pathway in irradiated tumor cells attenuated the proliferation of living tumor cells nearby in vitro

**Fig. 4:**
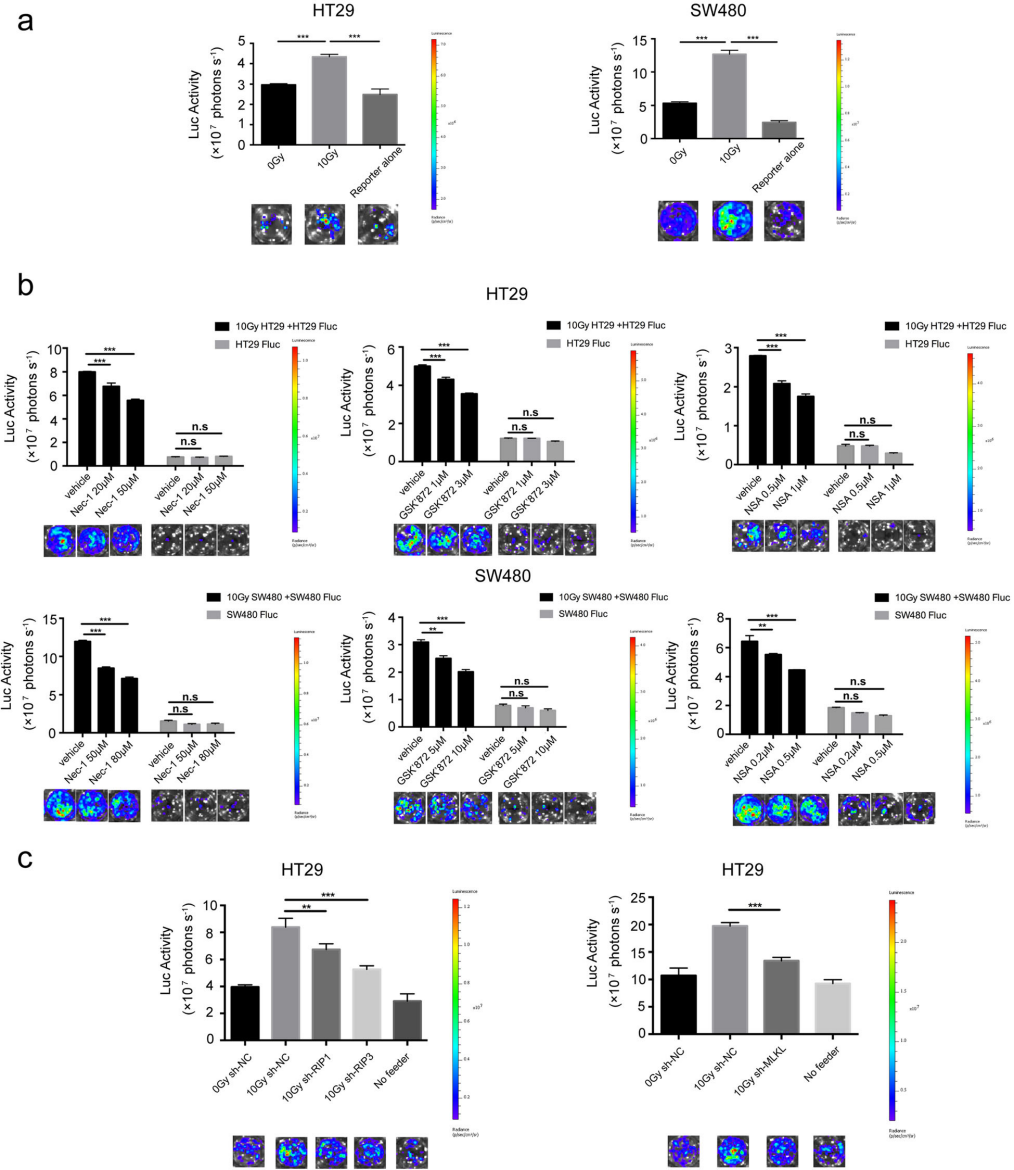
Blockade of the RIP1/RIP3/MLKL signaling pathway in irradiated tumor cells attenuated the proliferation of living tumor cells nearby in vitro*.* **a** 10Gy-irradiated tumor cells prompted the proliferation of living tumor cell. Upper panel, growth of HT29 Fluc or SW480 Fluc were observed by luciferase activity imaging. Lower panel, representative bioluminescence images, one-way ANOVA, *n* = 3. **b** Proliferation-promoting effect of dying HT29 and SW480 cells on HT29 Fluc and SW480 Fluc in vitro. The dying cells pretreated with RIP1 inhibitor (Nec-1), RIP3 inhibitor (GSK’872) or MLKL inhibitor (NSA) for 24 h, one-way ANOVA, *n* = 3. **c** Proliferation-promoting effect of dying sh-RIP1 HT29 cells, sh-RIP3 HT29 cell and sh-MLKL HT29 cells on HT29 Fluc cells, compared with equally treated vector-transfected HT29 cells, one-way ANOVA, *n* = 3. n.s = not significant, ** *p* < 0.005, *** *p* < 0.001

We next examined the effect of necroptotic cells on surrounding living cells. We decided to use bioluminescence to carry out quantitation of cell numbers. We first demonstrated that bioluminescence values (photons) were linearly related to the cell numbers of the luciferase-GFP gene labeled living cells (HT29 Fluc, SW480 Fluc and HCT116 Fluc, also called reporter cells) (Additional file 1: Figure S5). We employed a co-culture repopulation model to examine whether irradiated necroptotic colorectal cancer cells promote the proliferation of living tumor cells, which was described in our previous studies [8]. Our results showed that the 10 Gy-irradiated dying cells (irradiated HT29 and SW480 cells, feeder cells) strongly stimulated the proliferation of the HT29 Fluc and SW480 Fluc cells (reporter cells) (Fig. 4a), which could be significantly attenuated by RIP1, RIP3 and MLKL inhibitors (Fig. 4b). Notably, the inhibitors of RIP1, RIP3 and MLKL at certain concentrations could abolish the growth-stimulating effect of dying cells, but with no influence on the proliferation ability of HT29 Fluc cells or SW480 Fluc cells alone. To further confirm this observation, HT29 Fluc cells were seeded with irradiated HT29 sh-RIP1, HT29 sh-RIP3 and HT29 sh-MLKL cells and the growth of HT29 Fluc cells were weakened when compared with equally irradiated HT29 sh-NC cells as feeder (Fig. 4c). Additionally, to compare the growth-stimulating effect of irradiated HT29 cells and HCT116 cells, we employed the same number of feeder cells (irradiated HT29 cells and HCT116 cells) and reporter cells (HCT116 Fluc cells). The result showed that the growth-stimulating effect of irradiated HCT116 cells was significantly weakened than equally irradiated HT29 cells (Additional file 1: Figure S6). Altogether, these results demonstrated that necroptotic cells after irradiation indeed associated with the promotion of living tumor cell proliferation in vitro*.*

### The knockdown of MLKL inhibits the growth stimulation effect and tumorigenicity in vivo

**Fig. 5:**
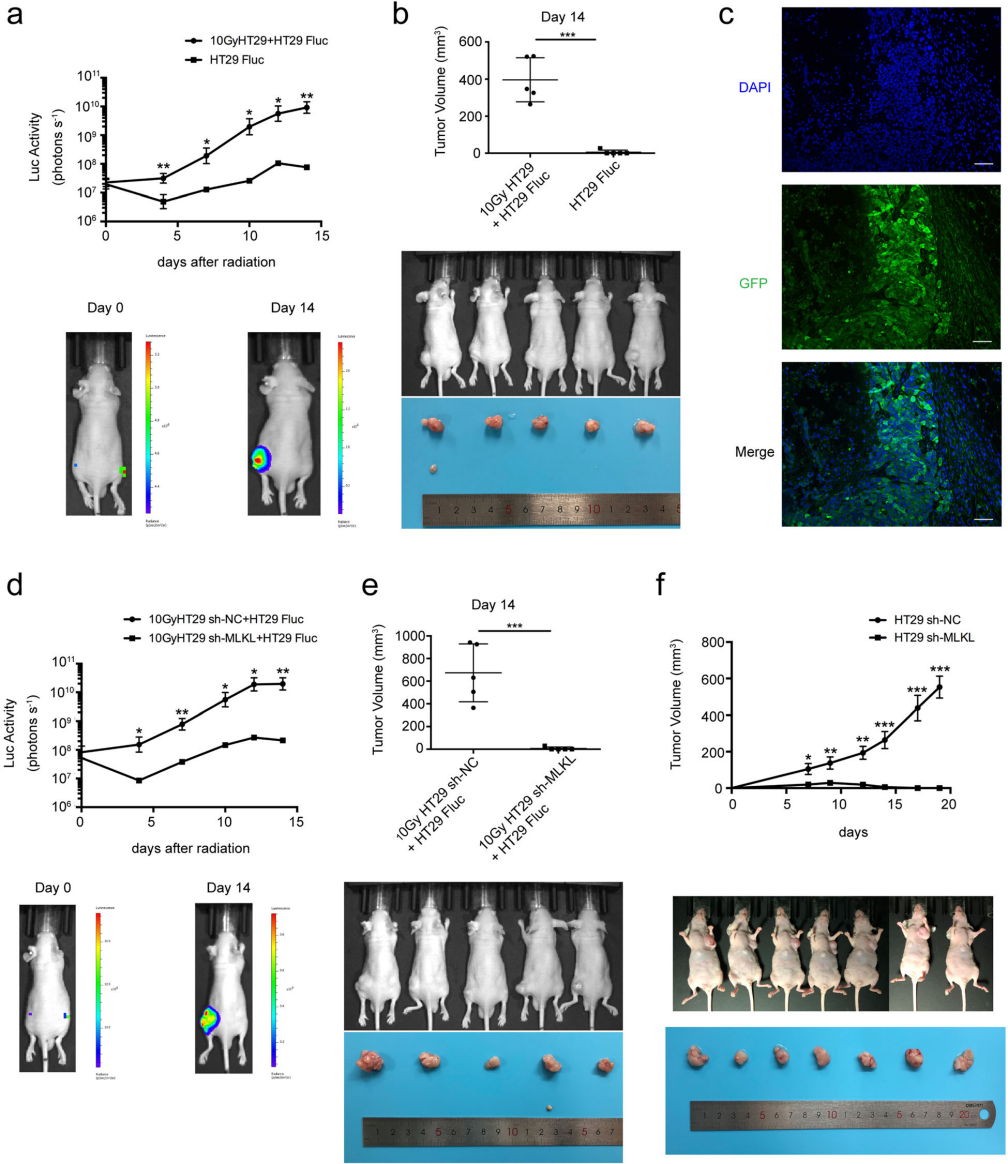
Knockdown of MLKL inhibited the growth stimulation effect and tumorigenicity in vivo*.* **a**–**c** Effect of dying HT29 on growth of HT29 Fluc in vivo. **a** Upper panel, proliferation of HT29 Fluc in vivo was monitored by bioluminescence imaging, student’s t-test, *n* = 5, lower panel, representative bioluminescent images of mice on Day 0 and Day 14. **b** Tumor volume of mice (upper panel) and photograph of xenograft (lower panel) on Day 14, student’s t-test, *n* = 5. **c** Immunofluorescence analysis showed GFP expression in HT29 tumor. Scale bar: 50 μm. **d**, **e** Effect of MLKL knockdown in dying HT29 cells on growth of HT29 Fluc in vivo. **d** Upper panel, proliferation of HT29 Fluc in vivo was monitored by bioluminescence imaging, student’s t-test, *n* = 5. Lower panel, representative bioluminescent images of mice on Day 0 and Day 14. **e** Tumor volume of mice (upper panel) and photograph of xenograft (lower panel) on Day 14, student’s t-test, *n* = 5. **f** Upper panel, xenograft tumor growth in nude mice from HT29 vector-transfected cells and HT29 sh-MLKL cells, student’s t-test, *n* = 7. Lower panel, Photograph of nude mice bearing HT29 vector-transfected and HT29 sh-MLKL xenograft. * < *p* 0.05, ** *p* < 0.005, *** *p* < 0.001

As shown in Fig. 5a and b, the irradiated HT29 markedly promoted the growth of HT29 Fluc (left hind legs) in vivo when compared with an equal number of HT29 Fluc alone (right hind legs)*.* To verify the growth of tumor cells in vivo was mainly from HT29 Fluc, we conducted immunofluorescence staining for GFP which was fused with Fluc. Figure 5c indicated that almost all cells in tumor mass were GFP-positive cells i.e. tumor mass derived from HT29Fluc cells. Next we further explored the role of necroptosis in dying cell stimulated tumor cell proliferation in vivo*.* Previous studies have demonstrated that MLKL is the critical downstream mediator of RIP1/RIP3 during radiation-induced necroptosis. We observed that the knockdown of MLKL in irradiated HT29 cells significantly reduced the growth of HT29 Fluc cells (right hind legs) in vivo, when compared with irradiated vector-transfected HT29 cells (left hind legs) (Fig. 5d and e). Interestingly, tumorigenicity experiments showed that there was no tumor formation in nude mice after knockdown of MLKL, in contrast to vector-transduced HT29 cells (Fig. 5f). Overall, these results demonstrate that the proliferation-promoting effect of radiation-induced dying cells as well as tumorigenicity in vivo were mediated by MLKL*.*

### JNK/IL-8 (CXCL8): a novel downstream axis of necroptosis in tumor repopulation

**Fig. 6:**
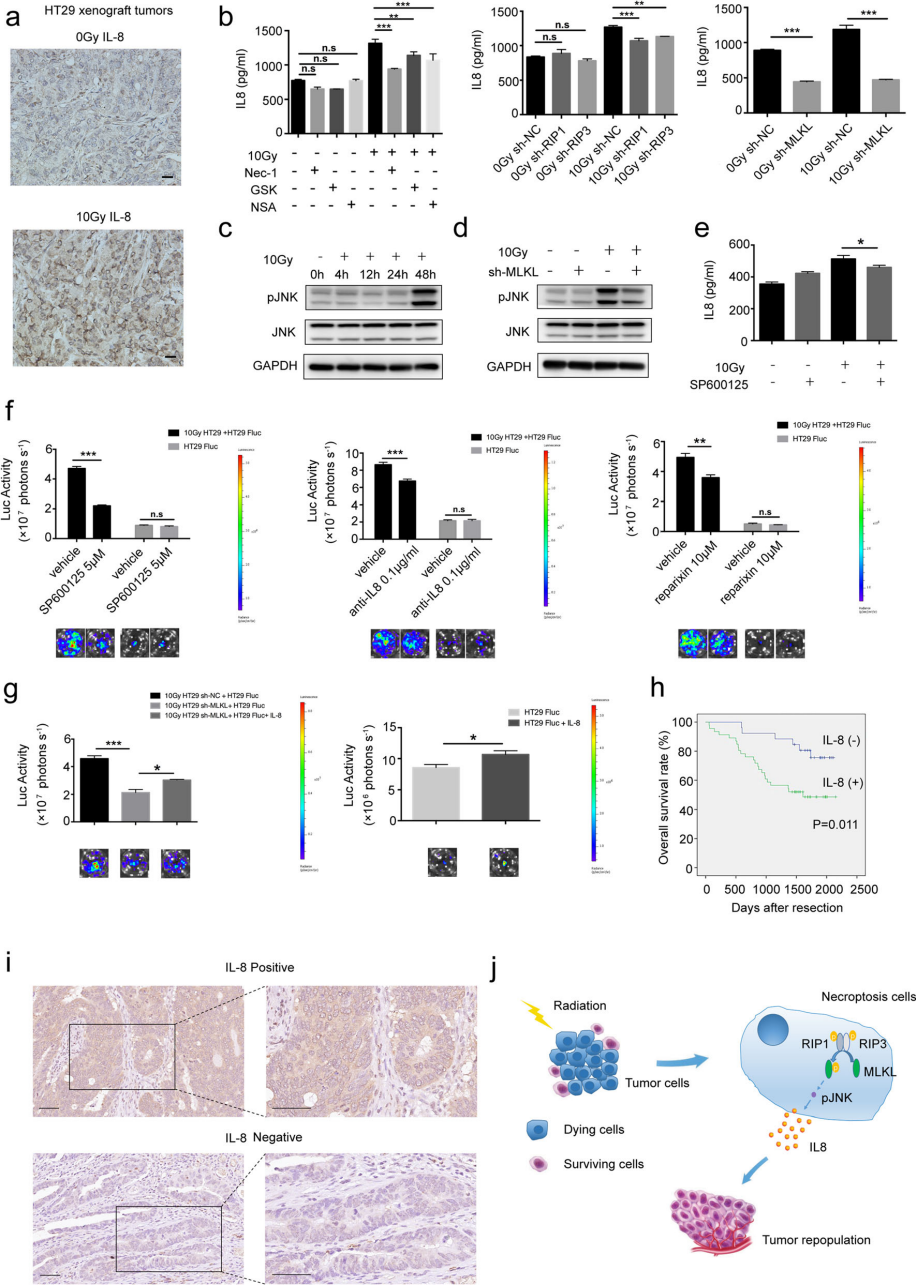
JNK/IL-8 (CXCL8): a novel downstream axis of necroptosis in tumor repopulation. **a** Immunohistochemical staining of IL-8 in the HT29 xenograft tumors for 48 h from 10 Gy-irradiated and no-irradiated nude mice. Scale bar: 20 μm. **b** Left panel, IL-8 concentrations were measured in the supernatants of HT29 cells (pretreated with RIP1 inhibitor (Nec-1), RIP3 inhibitor (GSK’872) or MLKL inhibitor (NSA) for 24 h) with no radiation or after 10Gy radiation for 48 h by chemiluminescent immunoassays. Right panel, IL-8 concentrations were measured in the supernatants of HT29 sh-NC, HT29 sh-RIP1, HT29 sh-RIP3 and HT29 sh-MLKL cells with or without 10 Gy radiation for 48 h, one-way ANOVA, *n* = 3. **c** Western blot showed the protein level of phosphorylated JNK1/2 and pan-JNK in HT29 cells after 10Gy radiation. **d** Western blot showed expression of phosphorylated JNK1/2 and pan-JNK in MLKL knockdown and vector-transfected HT29 cells for 48 h after 10 Gy radiation. **e** IL-8 concentrations were measured in the supernatants of HT29 cells (pretreated with JNK inhibitor (SP600125)) with no radiation or after 10 Gy radiation for 48 h by chemiluminescent immunoassays, one-way ANOVA, *n* = 3. **f** Effect of dying HT29 cells on growth of HT29 Fluc after treatment of JNK inhibitor (SP600125), anti-IL-8 antibody and reparixin (an inhibitor of IL-8 receptor CXCR1 and CXCR2), one-way ANOVA, *n* = 3. **g** Effects of recombinant Human IL-8 on dying HT29 sh-MLKL cells induced growth of HT29 Fluc (left panel) and on growth of HT29 Fluc alone (right panel), one-way ANOVA, *n* = 3. **h** The Kaplan–Meier survival analysis in 71 colorectal cancer patients. **i** Representative images of positive (upper panel) and negative (lower panel) IL-8 staining in human colorectal cancer tissues. Scale bar: 50 μm. **j** Schematic representation of the proposed mechanisms for necroptosis–mediated tumor cell repopulation. n.s = not significant, * < *p* 0.05, ** *p* < 0.005, *** *p* < 0.001

Recent studies have revealed that necroptosis plays an important role in inflammation, and indicate that necroptosis might produce pro-inflammatory cytokines [23–25]. We measured six pro-inflammatory cytokines level (TNF-α, IL-1β, IL-2R, IL-6, IL-8 and IL-10) in the supernatants of HT29 cells 48 h later after irradiation, and interestedly only IL-8 exhibited a higher background expression and a significant increase after irradiation. Next, we performed immunohistochemical staining to evaluate the expression of IL-8 in xenograft tumor in nude mice 48 h later after irradiation. As shown in Fig. 6a, the tumor tissue post irradiation showed significant increases in the expression of IL-8. Chemiluminescent immunoassays showed that the IL-8 concentration in the supernatants of irradiated HT29 sh-RIP1, HT29 sh-RIP3 and HT29 sh-MLKL cells was much lower than that in the control, and the similar results were observed after treatment with RIP1, RIP3 and MLKL inhibitors (Fig. 6b). Since it has been reported that activation of c-jun N-terminal kinase (JNK) could facilitate IL-8 production [26, 27], and MLKL plays a role on JNK activation [12]. Therefore, we investigated whether JNK was activated in irradiated dying cells. Western blot showed that 10 Gy irradiation markedly upregulated the phosphorylation of JNK1/2 (48 h post-radiation) in HT29 cells (Fig. 6c), however, 10 Gy-irradiated HT29 sh-MLKL cells did not show elevated level of phospho-JNK1/2 (Fig. 6d). Moreover, chemiluminescent immunoassays showed that inhibition of JNK activity by SP600125 also reduced radiation-induced IL-8 release (Fig. 6e), indicating that IL-8 might be regulated by JNK.

In order to confirm that JNK/IL-8 was the downstream signaling pathway in necroptosis-induced tumor repopulation, we conducted following experiments. As Fig. 6f showed, the proliferation promotion effect of irradiated HT29 cells was inhibited by using the JNK inhibitor SP600125 or an IL-8 neutralizing antibody, which had no effect on HT29 Fluc alone. In addition, a similar effect can be detected after treatment of reparixin, an inhibitor of the IL-8 receptors chemokine (C-X-C motif) receptor 1 (CXCR1) and chemokine (C-X-C motif) receptor 2 (CXCR2) (Fig. 6f). Finally, recombinant Human IL-8 treatment could rescue the growth-stimulating effect of irradiated HT29 sh-MLKL cells (Fig. 6g). Taken together, these results suggest that JNK/IL-8 plays an important role in necroptosis-mediated tumor repopulation in some colorectal cancer.

We next identified the clinical relevance of our newly discovered factors (IL-8). As has been previously reported, IL-8 is a cytokine secreted by macrophages. However, we found it could be secreted by colorectal cancer cells and further increased by X-ray irradiation. Therefore, we conducted immunohistochemistry staining for IL-8 in colorectal cancer tissue microarrays from 71 patients. Kaplan-Meier analyses showed that the elevated IL-8 expression was significantly correlated with poor overall survival (Fig. 6h). In Fig. 6i, representative images presented weak and strong expression of IL-8 in colorectal tumor tissues. Our results therefore suggest that IL-8 expression levels might be a new marker for prognosis in patients with CRC.

## Discussion

RT is an important modality for the treatment of colorectal cancer. RT dose is usually split into multiple parts, and the intervals are designed to allow adjacent normal tissues recovery. Unfortunately, the survived tumor cells also get chance to proliferate even at accelerated paces in the intervals during RT, which is called tumor repopulation. More importantly, tumor repopulation is one of the major causes of radioresistance and tumor recurrence, thus ultimately leading to treatment failure. However, the mechanism of tumor repopulation remains unclear.

The cell death has been recognized as the goal to treat the tumors. The radiation can induce cell death by causing nuclear DNA damage. The apoptosis has been reported to be a major manner of radiation induced cell death [28]. However, necrosis has also been reported to be associated with radiation induced cell death [29]. Recently, a novel cell death manner, necroptosis, has been reported. Since our previous studies and other reporters suggested that cell death is not a passive process, dying cells may play an active role to support nearby live cell proliferation via releasing numerous growth factors even they are repaired by themselves then grow as tumor stem like cells and finally caused tumor recurrence or tumor repopulation. In this study, we first demonstrated that radiation could also induce necroptosis. Like apoptosis and necrosis this novel cell death manner could also induce tumor cells nearby growth by upregulating the phosphorylation of RIP1 and RIP3, leading to the formation of the RIP1/RIP3 necrosome complex and subsequent phosphorylation of MLKL in some colorectal cancer cells. The blockage of necroptosis regulation genes RIP1/RIP3/MLKL, especially MLKL, by small chemical inhibitors or genetic depletion markedly attenuated tumor repopulation in in vitro and in vivo and even attenuated tumorigenicity in mice.

It is notable to mention that our experiments showed that necroptosis is a double-edged sword. Though it appeared counter-intuitive, several other studies have also reported that necroptosis could help tumor growth or metastasis. For example, Seifert et al. [30] showed that the necrosome promotes pancreas oncogenesis; RIP1 is reported to be an oncogenic diver in melanoma [31], colorectal cancer [32] and liver tumor formation [33]; and Strilic et al. [34] even proposed that necroptosis promotes pulmonary tumor cells metastasis.

How RIP1/RIP3/MLKL mediated tumor repopulation? In consideration that necroptosis is reported to be involved in the production of pro-inflammatory cytokines [23–25]. Therefore, we examined 6 common cytokines (IL-1β, IL-2R, IL-6, IL-8 IL-10 and TNF-α) and found colorectal cancer derived HT29 and SW480 cells expressed IL-8 and further increased after irradiation. Several studies have found that IL-8 may exert pro-tumoral function [35–37]. We used an antibody against IL-8 and inhibitors for IL-8 receptors further confirming that IL-8 took part into the necroptotic cells mediated tumor repopulation. More interestedly, IL-8 expression level was associated with JNK phosphorylation level, which was tightly correlated with MLKL expression level and phosphorylation, as shown in MLKL shRNA transduced HT29 cells. Therefore, we propose a novel pathway RIP1/RIP3/MLKL/JNK/IL-8 implicated in the necroptotic cells mediated tumor repopulation.

Taking together our findings provide a new insight into the function of MLKL molecule and the mechanism of tumor repopulation. However, we still do not know why activated MLKL was first transported into the nucleus and then translocated to cell membrane during necroptosis and more detailed characterization of RIP1/RIP3/MLKL/JNK/IL-8 pathway needs to be carried out.

Our findings suggest that activation of cell death-promoting factors (such as caspase 3 cleavage, HMGB1 release and MLKL phosphorylation) does not equal to immediate cell death even if these cells are destined to die. In their march to death, they would produce and release different growth–stimulating factors. These factors stimulate survived tumor cells nearby to proliferate and repopulate. As to which death mechanism the tumor cells would die is mainly determined by genetic alterations in each tumor cell. For instance, HT29, SW480 and HCT116 are all derived from colorectal cancer patients, but HCT116 did not show radiation induced necroptosis probably due to the lack of RIP3 expression. Interestedly, both HT29 and SW480 showed higher percentage of necroptosis than apoptosis or other cell death after irradiation, at least at the early stage post irradiation (Additional file 1: Figure S3). One limitation of this study is that it mainly focused on the paracrine effects of necropototic cells on survived tumor cells nearby. However, whether the necroptosis-bound cells themselves could rescue themselves remain unknown and could be a worthwhile subject for future studies.

## Conclusion

In conclusion, results from this study demonstrated that radiation-induced necroptosis depends on the activation of RIP1/RIP3/MLKL pathway, and the necroptosis contributes to tumor repopulation through the MLKL/JNK/IL-8 axis. Furthermore, elevated expression of IL-8 in tumor tissues predicts for a worse prognosis in colorectal cancer patients. Finally, MLKL/JNK/IL-8 could be potential targets for blocking tumor repopulation and improving the efficacy of radiotherapy.

## Supplementary information

**Additional file 1: Figure S1.** Western blot analysis of necroptotic factor in colorectal cancer cells. (a) The endogenous levels of RIP1, RIP3 and MLKL expression in HT29, SW480 and HCT116 cells were evaluated by Western blot. (b) Western bolt showed pMLKL (T357/S358) level in HT29 cells pre-treated with or without Nec-1 and irradiated or non-irradiated by 10 Gy X-ray. (c) Western blot showed the protein level of phosphorylated RIP1 (S166), phosphorylated RIP3 (S227), phosphorylated MLKL (T357/S358) in SW480 cells after 10 Gy irradiation. **Figure S2.** Clonogenic survival assays of irradiated HT29 and SW480 cells. (a) The picture of survived colonies of HT29 and SW480 cells irradiated by different dose of X-ray and seeded in different numbers. **Figure S3.** The cell death manners of HT29 and SW480 cells treated by inhibitors and radiation. (a) Representative graphs and statistical results of flow cytometry analyses after Annexin V/PI double staining. HT29 and SW480 cells were analyzed 3 days later after irradiation and inhibitor treatment as shown in Fig. Necroptosis was counted by the percentage of decreased PI positive cells by Nec-1. Apoptosis was counted by the percentage of decreased Annexin V positive cells by Z-vad-fmk. Ferroptosis was counted by the percentage of decreased of Annexin V negative/PI negative cells by Liproxstatin-1. One-way ANOVA, *n* = 3. ** *p* < 0.005, *** *p* < 0.001. **Figure S4.** Radiation-induced necroptosis depended on the RIP1/RIP3/MLKL signaling pathway. (a) The efficiency of knockdown of RIP1, RIP3 and MLKL by shRNA in HT29 cells were confirmed by western blot. (b) The knockdown of RIP1 by shRNA and its effect on LDH release in HCT116 cells after irradiation was quantified, one-way ANOVA, *n* = 3. (c) The necroptosis was further confirmed in irradiated or non-irradiated HT29 cells by using MLKL inhibitor (NSA) or shRNAs, one-way ANOVA, *n* = 3. n.s = not significant. **Figure S5.** Correlation between Luc activity and Luc gene labeled cell numbers. The measured luciferase activity (HT29 Fluc, SW480 Fluc and HCT116 Fluc) showed linearity with the plated cell numbers. **Figure S6.** Proliferation-promoting effect of irradiated HT29 and HCT116 cells on HCT116 Fluc in vitro*.* HCT116 Fluc cells showed differential growth on irradiated HT29 and HCT116 cells*,* one-way ANOVA, *n* = 3*.* * *p* < 0.05, *** *p* < 0.001. **Table S1.** Expression of IL-8 and clinic-pathologic features in colorectal cancer patients.

## Data Availability

The data used and analyzed during this study are available from the corresponding author on request.

## Abbreviations

CXCR1: Chemokine (C-X-C motif) receptor 1
CXCR2: Chemokine (C-X-C motif) receptor 2
JNK: c-jun N-terminal kinase
MLKL: Mixed lineage kinase domain-like protein
PGE2: Prostaglandin E2
RIP1: Receptor interacting protein 1
RIP3: Receptor interacting protein 3
