# Supplementary figures and images for: Necroptosis regulates tumor repopulation after radiotherapy via RIP1/RIP3/MLKL/JNK/IL8 pathway
Source: J Exp Clin Cancer Res. 2019 Nov 9;38:461. doi: 10.1186/s13046-019-1423-5 (PMC6842489; doi:10.1186/s13046-019-1423-5)

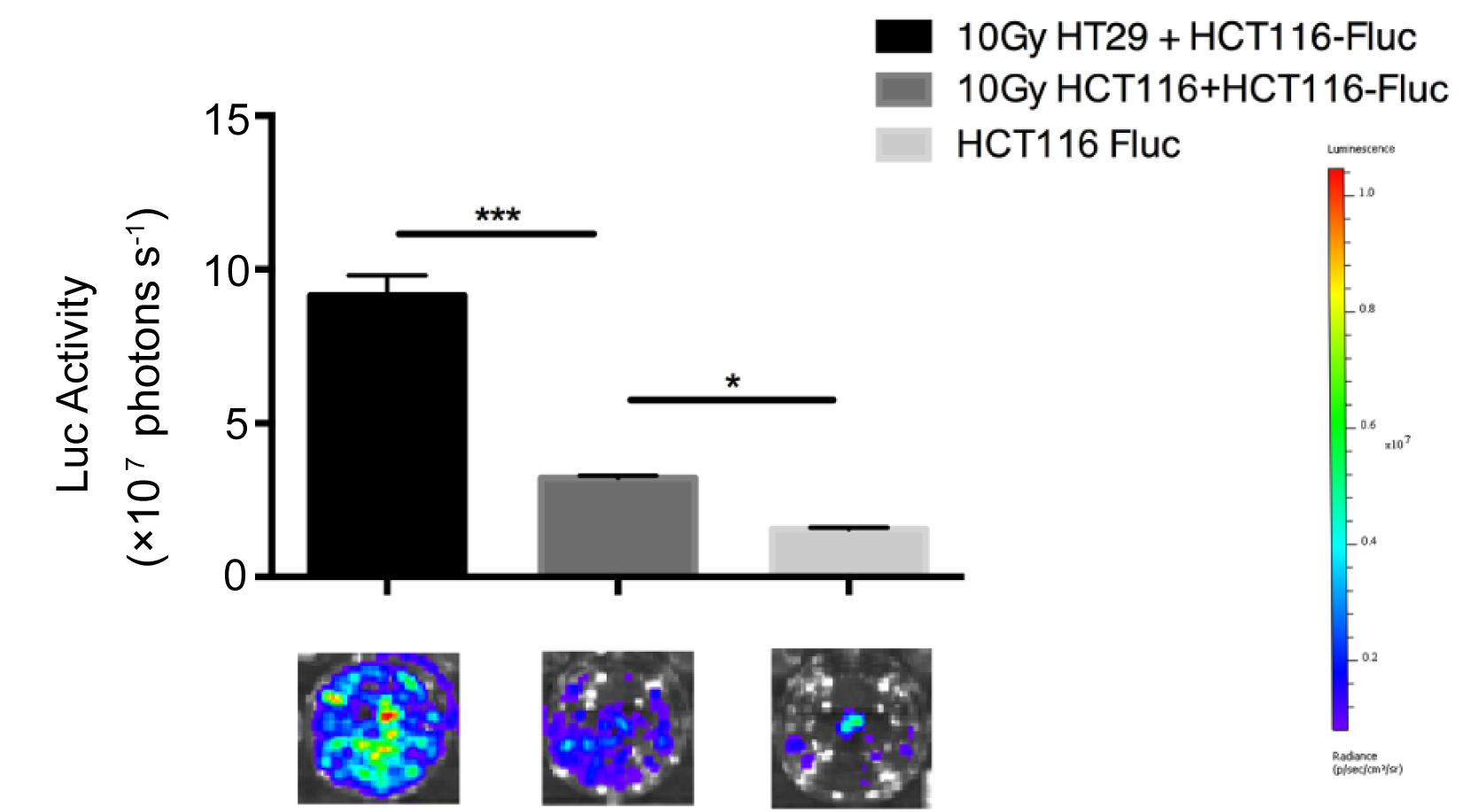

Supplement: Supplementary file 1 — Additional file 1: Figure S1. Western blot analysis of necroptotic factor in colorectal cancer cells. (a) The endogenous levels of RIP1, RIP3 and MLKL expression in HT29, SW480 and HCT116 cells were evaluated by Western blot. (b) Western bolt showed pMLKL (T357/S358) level in HT29 cells pre-treated with or without Nec-1 and irradiated or non-irradiated by 10 Gy X-ray. (c) Western blot showed the protein level of phosphorylated RIP1 (S166), phosphorylated RIP3 (S227), phosphorylated MLKL (T357/S358) in SW480 cells after 10 Gy irradiation. Figure S2. Clonogenic survival assays of irradiated HT29 and SW480 cells. (a) The picture of survived colonies of HT29 and SW480 cells irradiated by different dose of X-ray and seeded in different numbers. Figure S3. The cell death manners of HT29 and SW480 cells treated by inhibitors and radiation. (a) Representative graphs and statistical results of flow cytometry analyses after Annexin V/PI double staining. HT29 and SW480 cells were analyzed 3 days later after irradiation and inhibitor treatment as shown in Fig. Necroptosis was counted by the percentage of decreased PI positive cells by Nec-1. Apoptosis was counted by the percentage of decreased Annexin V positive cells by Z-vad-fmk. Ferroptosis was counted by the percentage of decreased of Annexin V negative/PI negative cells by Liproxstatin-1. One-way ANOVA, n = 3. ** p < 0.005, *** p < 0.001. Figure S4. Radiation-induced necroptosis depended on the RIP1/RIP3/MLKL signaling pathway. (a) The efficiency of knockdown of RIP1, RIP3 and MLKL by shRNA in HT29 cells were confirmed by western blot. (b) The knockdown of RIP1 by shRNA and its effect on LDH release in HCT116 cells after irradiation was quantified, one-way ANOVA, n = 3. (c) The necroptosis was further confirmed in irradiated or non-irradiated HT29 cells by using MLKL inhibitor (NSA) or shRNAs, one-way ANOVA, n = 3. n.s = not significant. Figure S5. Correlation between Luc activity and Luc gene labeled cell numbers. The mea [file 13046_2019_1423_MOESM1_ESM.zip › Fig. s6.jpg]

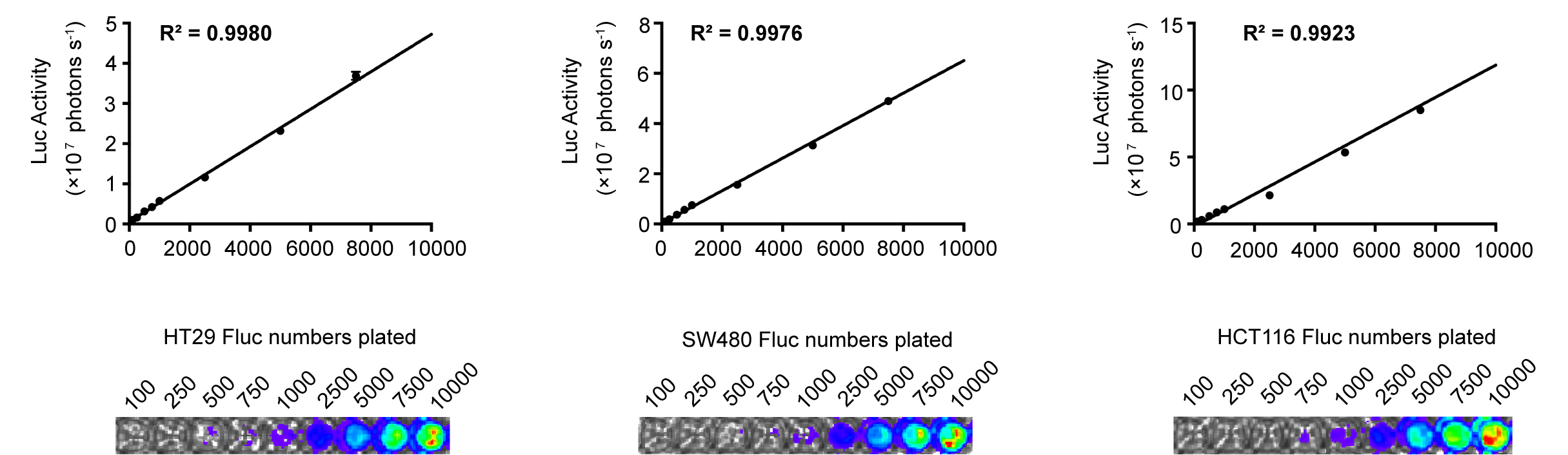

Supplement: Supplementary file 1 — Additional file 1: Figure S1. Western blot analysis of necroptotic factor in colorectal cancer cells. (a) The endogenous levels of RIP1, RIP3 and MLKL expression in HT29, SW480 and HCT116 cells were evaluated by Western blot. (b) Western bolt showed pMLKL (T357/S358) level in HT29 cells pre-treated with or without Nec-1 and irradiated or non-irradiated by 10 Gy X-ray. (c) Western blot showed the protein level of phosphorylated RIP1 (S166), phosphorylated RIP3 (S227), phosphorylated MLKL (T357/S358) in SW480 cells after 10 Gy irradiation. Figure S2. Clonogenic survival assays of irradiated HT29 and SW480 cells. (a) The picture of survived colonies of HT29 and SW480 cells irradiated by different dose of X-ray and seeded in different numbers. Figure S3. The cell death manners of HT29 and SW480 cells treated by inhibitors and radiation. (a) Representative graphs and statistical results of flow cytometry analyses after Annexin V/PI double staining. HT29 and SW480 cells were analyzed 3 days later after irradiation and inhibitor treatment as shown in Fig. Necroptosis was counted by the percentage of decreased PI positive cells by Nec-1. Apoptosis was counted by the percentage of decreased Annexin V positive cells by Z-vad-fmk. Ferroptosis was counted by the percentage of decreased of Annexin V negative/PI negative cells by Liproxstatin-1. One-way ANOVA, n = 3. ** p < 0.005, *** p < 0.001. Figure S4. Radiation-induced necroptosis depended on the RIP1/RIP3/MLKL signaling pathway. (a) The efficiency of knockdown of RIP1, RIP3 and MLKL by shRNA in HT29 cells were confirmed by western blot. (b) The knockdown of RIP1 by shRNA and its effect on LDH release in HCT116 cells after irradiation was quantified, one-way ANOVA, n = 3. (c) The necroptosis was further confirmed in irradiated or non-irradiated HT29 cells by using MLKL inhibitor (NSA) or shRNAs, one-way ANOVA, n = 3. n.s = not significant. Figure S5. Correlation between Luc activity and Luc gene labeled cell numbers. The mea [file 13046_2019_1423_MOESM1_ESM.zip › Fig. s5.jpg]

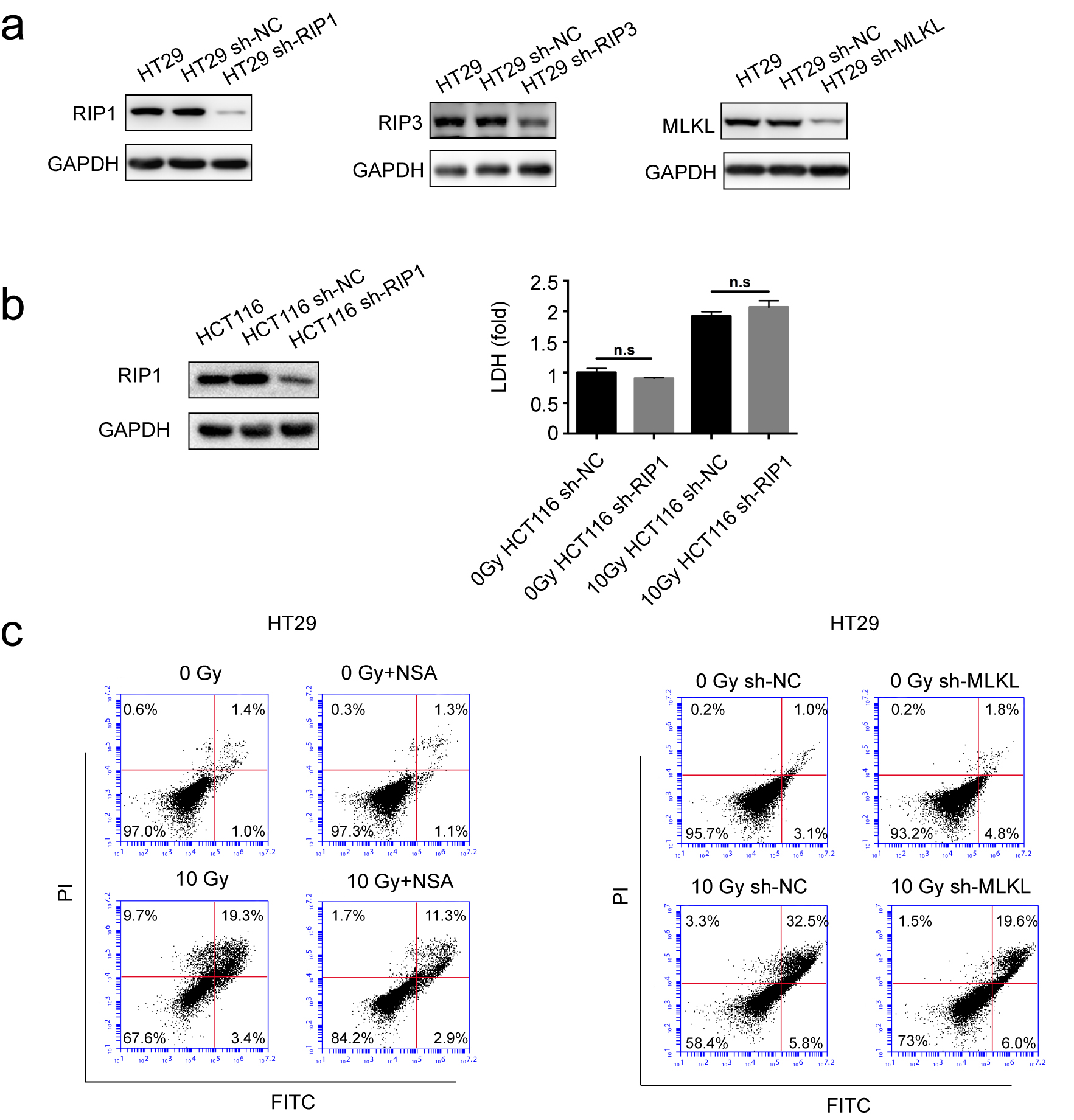

Supplement: Supplementary file 1 — Additional file 1: Figure S1. Western blot analysis of necroptotic factor in colorectal cancer cells. (a) The endogenous levels of RIP1, RIP3 and MLKL expression in HT29, SW480 and HCT116 cells were evaluated by Western blot. (b) Western bolt showed pMLKL (T357/S358) level in HT29 cells pre-treated with or without Nec-1 and irradiated or non-irradiated by 10 Gy X-ray. (c) Western blot showed the protein level of phosphorylated RIP1 (S166), phosphorylated RIP3 (S227), phosphorylated MLKL (T357/S358) in SW480 cells after 10 Gy irradiation. Figure S2. Clonogenic survival assays of irradiated HT29 and SW480 cells. (a) The picture of survived colonies of HT29 and SW480 cells irradiated by different dose of X-ray and seeded in different numbers. Figure S3. The cell death manners of HT29 and SW480 cells treated by inhibitors and radiation. (a) Representative graphs and statistical results of flow cytometry analyses after Annexin V/PI double staining. HT29 and SW480 cells were analyzed 3 days later after irradiation and inhibitor treatment as shown in Fig. Necroptosis was counted by the percentage of decreased PI positive cells by Nec-1. Apoptosis was counted by the percentage of decreased Annexin V positive cells by Z-vad-fmk. Ferroptosis was counted by the percentage of decreased of Annexin V negative/PI negative cells by Liproxstatin-1. One-way ANOVA, n = 3. ** p < 0.005, *** p < 0.001. Figure S4. Radiation-induced necroptosis depended on the RIP1/RIP3/MLKL signaling pathway. (a) The efficiency of knockdown of RIP1, RIP3 and MLKL by shRNA in HT29 cells were confirmed by western blot. (b) The knockdown of RIP1 by shRNA and its effect on LDH release in HCT116 cells after irradiation was quantified, one-way ANOVA, n = 3. (c) The necroptosis was further confirmed in irradiated or non-irradiated HT29 cells by using MLKL inhibitor (NSA) or shRNAs, one-way ANOVA, n = 3. n.s = not significant. Figure S5. Correlation between Luc activity and Luc gene labeled cell numbers. The mea [file 13046_2019_1423_MOESM1_ESM.zip › Fig. s4.jpg]

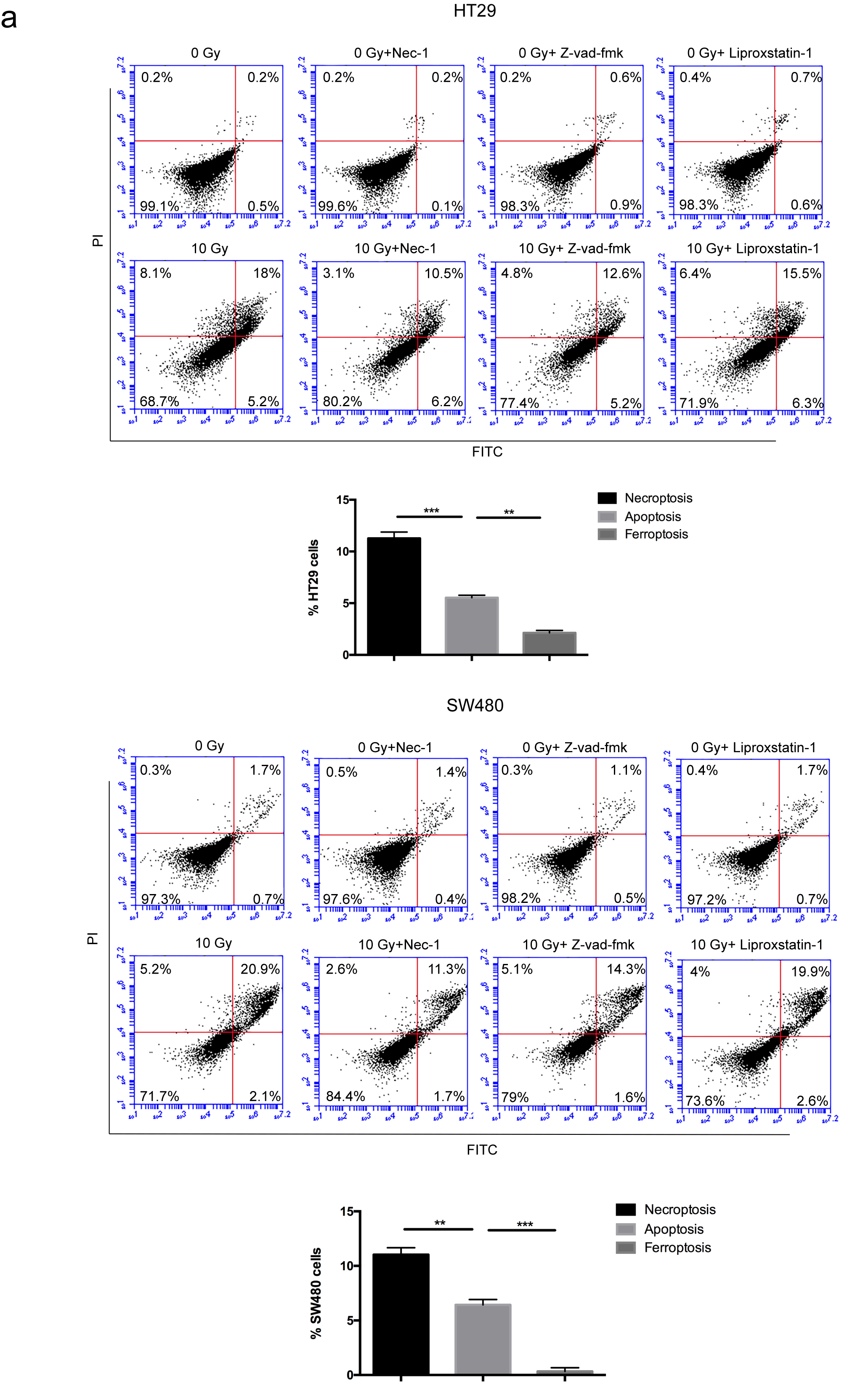

Supplement: Supplementary file 1 — Additional file 1: Figure S1. Western blot analysis of necroptotic factor in colorectal cancer cells. (a) The endogenous levels of RIP1, RIP3 and MLKL expression in HT29, SW480 and HCT116 cells were evaluated by Western blot. (b) Western bolt showed pMLKL (T357/S358) level in HT29 cells pre-treated with or without Nec-1 and irradiated or non-irradiated by 10 Gy X-ray. (c) Western blot showed the protein level of phosphorylated RIP1 (S166), phosphorylated RIP3 (S227), phosphorylated MLKL (T357/S358) in SW480 cells after 10 Gy irradiation. Figure S2. Clonogenic survival assays of irradiated HT29 and SW480 cells. (a) The picture of survived colonies of HT29 and SW480 cells irradiated by different dose of X-ray and seeded in different numbers. Figure S3. The cell death manners of HT29 and SW480 cells treated by inhibitors and radiation. (a) Representative graphs and statistical results of flow cytometry analyses after Annexin V/PI double staining. HT29 and SW480 cells were analyzed 3 days later after irradiation and inhibitor treatment as shown in Fig. Necroptosis was counted by the percentage of decreased PI positive cells by Nec-1. Apoptosis was counted by the percentage of decreased Annexin V positive cells by Z-vad-fmk. Ferroptosis was counted by the percentage of decreased of Annexin V negative/PI negative cells by Liproxstatin-1. One-way ANOVA, n = 3. ** p < 0.005, *** p < 0.001. Figure S4. Radiation-induced necroptosis depended on the RIP1/RIP3/MLKL signaling pathway. (a) The efficiency of knockdown of RIP1, RIP3 and MLKL by shRNA in HT29 cells were confirmed by western blot. (b) The knockdown of RIP1 by shRNA and its effect on LDH release in HCT116 cells after irradiation was quantified, one-way ANOVA, n = 3. (c) The necroptosis was further confirmed in irradiated or non-irradiated HT29 cells by using MLKL inhibitor (NSA) or shRNAs, one-way ANOVA, n = 3. n.s = not significant. Figure S5. Correlation between Luc activity and Luc gene labeled cell numbers. The mea [file 13046_2019_1423_MOESM1_ESM.zip › Fig. s3.jpg]

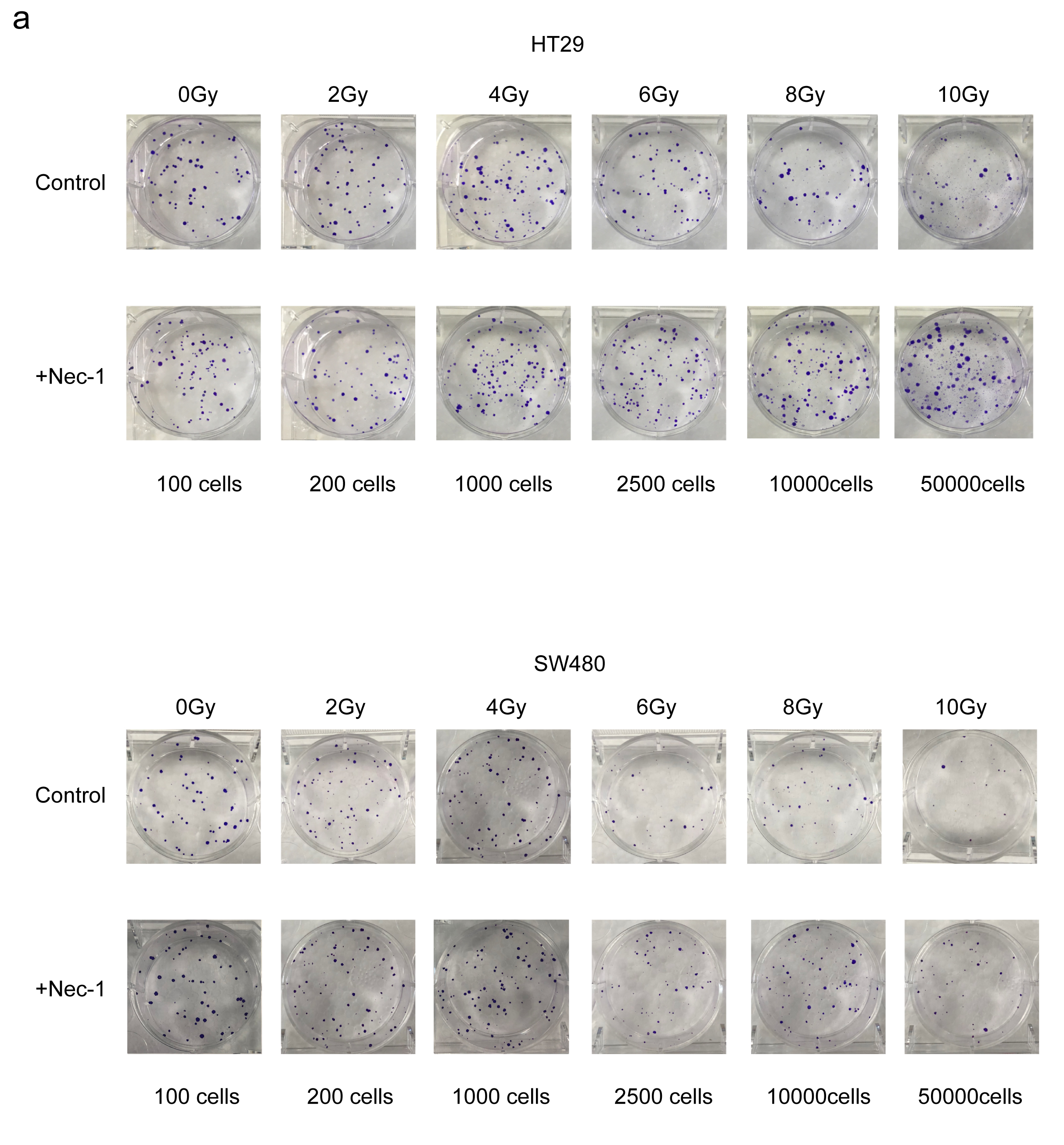

Supplement: Supplementary file 1 — Additional file 1: Figure S1. Western blot analysis of necroptotic factor in colorectal cancer cells. (a) The endogenous levels of RIP1, RIP3 and MLKL expression in HT29, SW480 and HCT116 cells were evaluated by Western blot. (b) Western bolt showed pMLKL (T357/S358) level in HT29 cells pre-treated with or without Nec-1 and irradiated or non-irradiated by 10 Gy X-ray. (c) Western blot showed the protein level of phosphorylated RIP1 (S166), phosphorylated RIP3 (S227), phosphorylated MLKL (T357/S358) in SW480 cells after 10 Gy irradiation. Figure S2. Clonogenic survival assays of irradiated HT29 and SW480 cells. (a) The picture of survived colonies of HT29 and SW480 cells irradiated by different dose of X-ray and seeded in different numbers. Figure S3. The cell death manners of HT29 and SW480 cells treated by inhibitors and radiation. (a) Representative graphs and statistical results of flow cytometry analyses after Annexin V/PI double staining. HT29 and SW480 cells were analyzed 3 days later after irradiation and inhibitor treatment as shown in Fig. Necroptosis was counted by the percentage of decreased PI positive cells by Nec-1. Apoptosis was counted by the percentage of decreased Annexin V positive cells by Z-vad-fmk. Ferroptosis was counted by the percentage of decreased of Annexin V negative/PI negative cells by Liproxstatin-1. One-way ANOVA, n = 3. ** p < 0.005, *** p < 0.001. Figure S4. Radiation-induced necroptosis depended on the RIP1/RIP3/MLKL signaling pathway. (a) The efficiency of knockdown of RIP1, RIP3 and MLKL by shRNA in HT29 cells were confirmed by western blot. (b) The knockdown of RIP1 by shRNA and its effect on LDH release in HCT116 cells after irradiation was quantified, one-way ANOVA, n = 3. (c) The necroptosis was further confirmed in irradiated or non-irradiated HT29 cells by using MLKL inhibitor (NSA) or shRNAs, one-way ANOVA, n = 3. n.s = not significant. Figure S5. Correlation between Luc activity and Luc gene labeled cell numbers. The mea [file 13046_2019_1423_MOESM1_ESM.zip › Fig. s2.jpg]

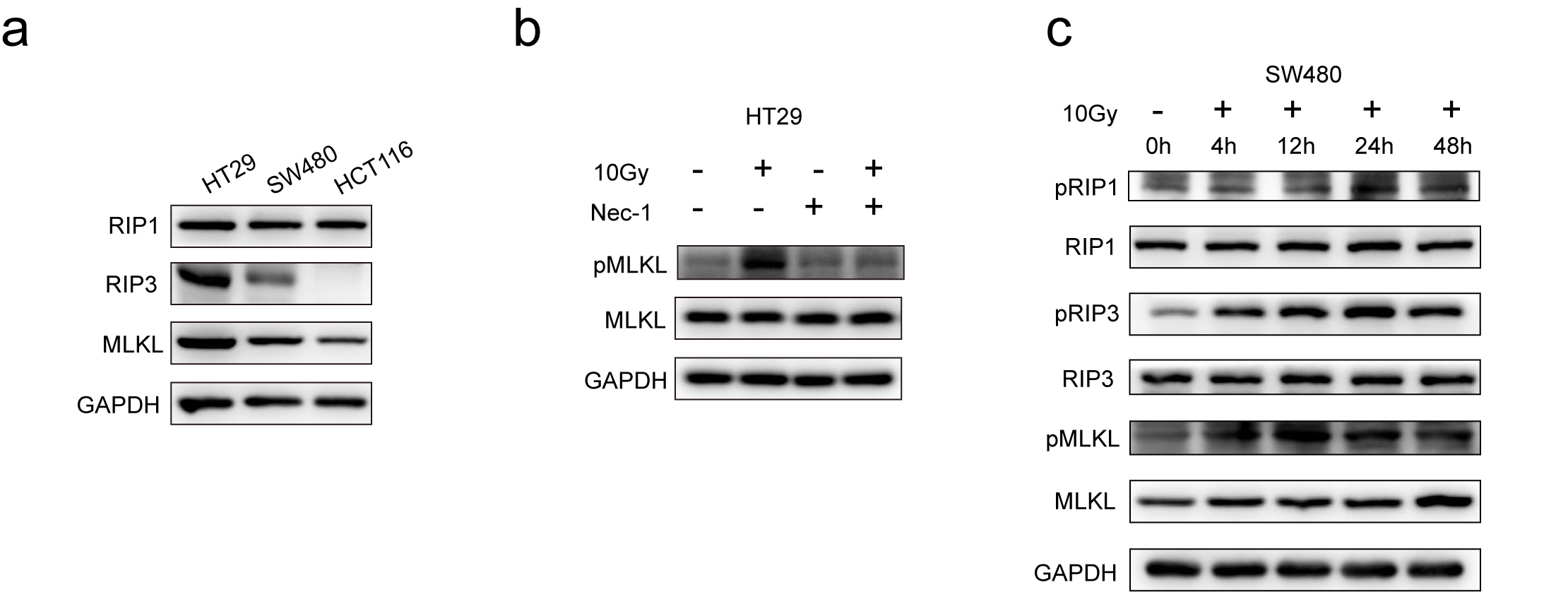

Supplement: Supplementary file 1 — Additional file 1: Figure S1. Western blot analysis of necroptotic factor in colorectal cancer cells. (a) The endogenous levels of RIP1, RIP3 and MLKL expression in HT29, SW480 and HCT116 cells were evaluated by Western blot. (b) Western bolt showed pMLKL (T357/S358) level in HT29 cells pre-treated with or without Nec-1 and irradiated or non-irradiated by 10 Gy X-ray. (c) Western blot showed the protein level of phosphorylated RIP1 (S166), phosphorylated RIP3 (S227), phosphorylated MLKL (T357/S358) in SW480 cells after 10 Gy irradiation. Figure S2. Clonogenic survival assays of irradiated HT29 and SW480 cells. (a) The picture of survived colonies of HT29 and SW480 cells irradiated by different dose of X-ray and seeded in different numbers. Figure S3. The cell death manners of HT29 and SW480 cells treated by inhibitors and radiation. (a) Representative graphs and statistical results of flow cytometry analyses after Annexin V/PI double staining. HT29 and SW480 cells were analyzed 3 days later after irradiation and inhibitor treatment as shown in Fig. Necroptosis was counted by the percentage of decreased PI positive cells by Nec-1. Apoptosis was counted by the percentage of decreased Annexin V positive cells by Z-vad-fmk. Ferroptosis was counted by the percentage of decreased of Annexin V negative/PI negative cells by Liproxstatin-1. One-way ANOVA, n = 3. ** p < 0.005, *** p < 0.001. Figure S4. Radiation-induced necroptosis depended on the RIP1/RIP3/MLKL signaling pathway. (a) The efficiency of knockdown of RIP1, RIP3 and MLKL by shRNA in HT29 cells were confirmed by western blot. (b) The knockdown of RIP1 by shRNA and its effect on LDH release in HCT116 cells after irradiation was quantified, one-way ANOVA, n = 3. (c) The necroptosis was further confirmed in irradiated or non-irradiated HT29 cells by using MLKL inhibitor (NSA) or shRNAs, one-way ANOVA, n = 3. n.s = not significant. Figure S5. Correlation between Luc activity and Luc gene labeled cell numbers. The mea [file 13046_2019_1423_MOESM1_ESM.zip › Fig. s1.jpg]
